# Bibliographical

**Published:** 1872-05

**Authors:** 


### BIBLIOGRAPHICAL.

*A Practical Treatise on the Diseases of Women.* By T. GAILLARD THOMAS, M.D., Professor of Obstetrics and Diseases of Women and Children in the College of Physicians and Surgeons, New York; Physician to the Roosevelt Hospital, New York, etc. Third Edition, Enlarged and Thoroughly Revised, with Two Hundred and Forty-Six Illustrations on Wood. Published by Henry C. Lea, Philadelphia.

The first edition of this work appeared in 1868, and although large, a second was demanded in the following year, and in two years, still another was called for.

The additions to the present volume amount to nearly one-fourth, and many portions have been rewritten, and several new chapters introduced. The author confesses that there has been some modifications in his views upon some points, but hopes this is an evidence of improved knowledge.

This already standard work simply requires an announcement, occupying, in our opinion, the advance in works on uterine therapeutics.

*A Treatise on Human Physiology.* Designed for the Use of Students and Practitioners of Medicine. By JNO. C. DALTON, M.D., Professor of Physiology and Hygiene in the College of Physicians and Surgeons, New York, etc., etc. Fifth Edition, Revised and Enlarged, with Two Hundred and Eighty-Four Illustrations. Henry C. Lea, Publisher, Philadelphia.

The general plan and arrangement of the previous editions have been preserved in this, but the entire work has been carefully revised, and many of its parts modified and rearranged. The constant advance of the natural and physical sciences has furnished many valuable aids to the physiologist, and in the language of the author, "Nothing is regarded of any value in natural science which is not based upon direct and intelligible observation or experiment; and accordingly, the discussion of doubtful or theoretical questions has been avoided, as a general rule, in the present volume, while new facts, from whatever source, if fully established, have been added and incorporated with the results of previous investigation. A number of new illustrations have been introduced, and a few of the older ones, which seem to be no longer useful, have been omitted. In all the changes and additions thus made, it has been the aim of the writer to make the book, in its present form, a faithful exponent of the actual condition of physiological science."

We presume that no more practical work on the subject can be found, or one combining so thoroughly the wants of the student and practitioner.

*An Introduction to Pathology and Morbid Anatomy.* By T. Henry Green, M.D., London, Member of the Royal College of Physicians; Lecturer on Pathology and Morbid Anatomy at Charing Cross Hospital Medical School, etc. Illustrated by Numerous Engravings on Wood. Philadelphia: Henry C. Lea, Publisher.

This work is designed to be strictly elementary, and is mainly intended as a text-book for students, and is, therefore, confined to a brief statement of the more important processes which take place in the human body, in accordance with the most modern views of the best pathologists. Discussion in regard to disputed points in pathology has been, to a great extent, avoided, and that view has been adopted which is sustained by the greatest weight of testimony. Recommended specially to students of medicine, or to practicing physicians who have not time to study more elaborate works.

*Pulmonary Consumption: Its Nature, Varieties and Treatment.* With an Analysis of One Thousand Cases to Exemplify its Duration. By C. J. B. Williams, M.D., F.R.S., Fellow of the Royal College of Physicians, Senior Consulting Physician to the Hospital for Consumption, Brompton, etc., and Chas. Theodore Williams, M.A., M.D., Oxon, Fellow of the Royal College of Physicians, and Physician to the Hospital for Consumption, Brompton. Henry C. Lea, Philadelphia.

This work is by the author of the "Principles of Medicine," who, during a period of nearly fifty years, has given special attention to the study of pulmonary consumption, under the tuition, while young, of such men as Alison, Laennec, Andral and Chomel, and afterward in connection with St. George's and University College Hospitals, for a period of twenty years. He speaks encouragingly of the present advance in the treatment of consumption, and hopefully for still further success in the future.

We have, also, from the same publisher, H. C. Lea, Philadelphia, in one handsome octavo volume of six hundred and twenty pages—

*A Complete Practical Treatise on the Diseases of Children.* By J. LEWIS SMITH, M. D., Professor of Morbid Anatomy in the Bellevue Hospital Medical College, New-York.

This is regarded, by the highest authorities, as one of the very best books upon the diseases of children, and as such, we commend it to the profession.

*A Clinical Manual of the Diseases of the Ear.* By LAWRENCE TURNBULL, M.D., Physician to the Department of Diseases of the Eye and Ear of Howard Hospital, of Philadelphia; Permanent Member and Corresponding Secretary of the Medical Society of the State of Pennsylvania; Permanent Member of the American Medical Association; Author of "Lectures on the Electro-Magnetic Telegraph," "The Nature, Causes and Treatment of Nervous Deafness," etc., etc. With a Colored Lithographic Plate, and over One Hundred Illustrations on Wood. Philadelphia: J. B. Lippincott & Co.

Diseases of the ear have ever been a source of great annoyance to the general practitioner. We have neither time or space to discuss the question as to whether the obscurity of these diseases is necessary or accidental, but would be permitted to say, that we feel confident that much of our ignorance is the result of the system adopted by our medical schools, in the majority of which, up to a few years past, this subject was treated as one of minor importance—or rather, in many instances, not treated at all. It is essential that physicians should be sufficiently informed to distinguish between the simple and more serious affections of this organ, so that when the latter present themselves, if they do not feel disposed or prepared to treat such cases, they will be able to direct them to some professional friend, where the necessary treatment can be administered.

Dr. Turnbull's book will be of great service to all who propose to keep pace with the progress in this department of our profession. Every physician should have it. Diseases of the ear have not received that attention by the profession that their importance demands, and are certainly a source of great annoyance to the general practitioner. However perfect his information in other departments of his profession, he consoles himself for his short-comings by the admitted obscurity of this class of diseases—often dismissing alike the most simple as well as the most serious diseases of this organ with some simple routine prescription, without an effort at examination, further than the statement of the patient—thus, in some cases, permitting a disease which in its incipiency yields readily to remedies, to extend itself, until there is the complete loss of the functions of this important organ.

*Earth as a Topical Applicatian in Surgery.* Being a full Exposition of its Uses in all the Cases Requiring Topical Applications Admitted in the Men's and Women's Surgical Wards of the Pennsylvania Hospital, During a Period of Six Months in 1869. By ADDINEL HEWSON, M.D., one of the Attending Surgeons to the Pennsylvania Hospital. With four Photo-Relief Illustrations. Lindsay & Blakiston, Philadelphia, through M. Lynch, Book-Seller, Atlanta, Georgia.

The perusal of this little work of three hundred pages has given us much pleasure. We commenced the reading greatly prejudiced against the idea of covering up wounds, mutilated limbs, and filling up suppurating cavities, with earth. A hasty review, however, of the ninety-two carefully recorded cases treated in the surgical wards of the Pennsylvania Hospital—embracing, as they do, the various wounds, surgical accidents, etc., usually met with in the surgical wards of large hospitals—has been sufficient to convince us that in certain conditions and surroundings, the earth-dressing is the very best plan of treatment. The result of this dressing in a number of the cases reported, is evidence positive of some of the advantages of earth applications over all others in certain conditions.

If Dr. Hewson can only obtain a hearing through his book, he will no longer be subjected to that ridicule which was so rife in certain localities some time ago.

Dr. Hewson gives one hundred pages to the discussion of the *modus operandi* of this treatment, claiming that its beneficial results are not alone due to the mere physical properties—such as excluding air, absorbing moisture and the excess of discharge, and affording uniform compression and support—but is, to a marked extent, due to other results. We will not, however, at this time, attempt a discussion of the theories advanced by Dr. Hewson.

In concluding this short notice, we would advise every member of the profession to procure this little work, as we know they will be fully compensated by its study.

*History of Medicine from the Earliest Ages to the Commencement of the Nineteenth Century.* By ROBLEY DUNGLISON, M.D., LL.D., Late Professor of the Institutes of Medicine and Medical Jurisprudence in the Jefferson Medical College, etc. Arranged and Edited by RICHARD J. DUNGLISON, M.D. Published by Messrs. Lindsay & Blakiston, Philadelphia. 1872.

This is a handsome volume of two hundred and eighty-seven pages, and is an embodiment of the course of lectures delivered at the University of Virginia in those palmy days when the distinguished author taught anatomy, surgery, the history of the progress and theories of medicine, physiology, materia medica, and pharmacy. It is "a condensed history of the progress of medicine, presenting all the main facts in systematic order—avoiding, as much as possible, prolixity or unnecessary discussion of the merits of men and theories, and not laying any claim whatever to the title of an exhaustive treatise." The work is divided into twenty-two chapters, on the

Origin of Medicine.

Medicine of the Ancient Egyptians.

Medicine of the Ancient Greeks.

Medicine of the Romans to the Time of Cato the Censor.

Medicine of the Jews up to the Captivity of Babylon.

Medicine of the Hindoos.

Medicine of the Chinese and Japanese.

Medicine of the Scythians.

Medicine of the Celts.

First Traces of a Medical Theory in the Philosophic Schools of Greece.

The Age of Hippocrates.

The Immediate Successors of Hippocrates.

The Empirical School.

State of Medicine Near the Dawn of the Christian Era.

State of Medicine During the Early Centuries of the Christian Era.

State of Medicine in Europe and the East from the Middle of the Sixth Century.

State of Medicine Among the Monks of the Middle Ages.

State of Medicine During the Thirteenth and Fourteenth Centuries.

State of Medicine During the Fifteenth Century.

State of Medicine During the Sixteenth Century.

State of Medicine During the Seventeenth Century.

State of Medicine During the Eighteenth Century.

The work concludes with the following appropriate and cheerful observation:

"When we take a retrospective glance at the condition of medicine in former times, and reflect upon the amount of ignorance, credulity and superstition that prevailed, we can not fail to be struck with the immense improvement that has taken place in comparatively modern periods, and must be encouraged in the hope, that as the physical and moral sciences pursue their onward progress, and as the means of observation and experiment are augmented and facilitated, our own noble science may attain a pitch of perfection of which, at the present time, we can form no adequate conception—shedding light where all is now obscurity, and tending to dispel doubt and difficulty wherever existent."

It is commended to the profession as containing all the essential facts in a brief space—carefully, intelligently and methodically arranged.

We also note from the same publishers, Messrs. Lindsay & Blakiston, Philadelphia—

*Memoranda on Poisons.* By the Late THOMAS HAWKS TANNER, M.D., F.L.S. Third and Completely Revised Edition.

This edition of Dr. Tanner's valuable "Memoranda on Poisons" varies somewhat from the original idea of furnishing simply or mainly to the practitioner a useful guide in cases of poisoning, in the incorporation of a larger amount of chemical knowledge—thus fitting it better for the student of medicine, though still retaining to a sufficient extent its practical bearing.

We have, also, from Messrs. Lindsay & Blakiston—

*Dr. Riyby's Obstetric Memoranda.* Fourth Edition, Revised and Enlarged. By ALFRED MEADOWS, M.D., Physician to the General Lying-in-Hospital and to the Hospital for Women; Author of a "Manual of Midwifery," etc.

This valuable little book contains, in separate chapters, practical observations upon pregnancy, natural parturition, obstetric operations, unnatural labor, complex labor, and puerperal diseases. The fact that three large editions of this little work have been exhausted is conclusive proof of its appreciation by the profession. It contains a large amount of valuable knowledge in a very small compass; and though some object to manuals, we have to say, let us have all the knowledge possible, in the most convenient and accessible form, whether it be in books great or small.

The editor (Dr. Meadows) has altered somewhat the previous arrangement—has added some new matter and omitted a portion of the old, by which he claims that he has more fully executed the original idea of Dr. Rigby, and thus made the book more practically useful.

These two little manuals can be readily carried in the pocket, and may thus aid, in a valuable manner, many a defective memory, and possibly save many a valuable life.

We also received (but overlooked) some time since, in connection with other books received at the time—

*Neuralgia, and the Diseases that Resemble It.* By FRANCIS E. ANSTIE, M.D., London, Fellow of the Royal College of Physicians, etc., etc. D. Appleton & Co., 549 and 551 Rroadway, New York. 1872.

The present work does not profess to be a mere compilation of what is contained in the various standard works, or scattered through current medical literature, but puts forward a claim to new views, first announced by the same author in "Reynolds' System of Medicine." The book contains:

An Introduction on Pain in General.

PART I.—On Neuralgia, with its Clinical History, its Complications, Pathology, Etiology, Diagnosis, Prognosis, and Treatment.

PART II.—Diseases that Resemble Neuralgia:—Myalgia; Spinal Irritation; The Pains of Hypochondriasis; The Pains of Locomotor Ataxy; The Pains of Cerebral Abscess; The Pains of Alcoholism; The Pains of Syphilis; The Pains of Subacute and Chronic Rheumatism; The Pains of Latent Gout, Colic, and Other Pains of Peripheral Irritation and Dyspeptic Headache.

The principal object in writing the volume is declared to be, to vindicate for neuralgia that distinct and independent position which the author has been long convinced it holds, and to show that it is not a mere offshoot of the gouty or rheumatic diathesis, or a symptom of a score of other diseases. In the author's own language, it is stated that, "In order to set the diagnosis of true neuralgia from its counterfeit in the clearest light, it seemed advisable to draw separate pictures of each of the latter (at least, of as many as were of real importance), and present them separately, as a kind of gallery of spurious neuralgias."

Though we have not yet had an opportunity of reading it, from a casual examination, we are satisfied that it contains, in a small compass, a large amount of curious, instructive and practical matter.

*Electricity in the Treatment of Diseases of the Skin.* By GEORGE M. BEARD, M.D., of New-York.

This little pamphlet is a reprint from the *American Journal of Syphilography and Dermatology*, and is intended to call the attention of the profession to the importance of electro-therapeutics in this class of diseases. Dr. Beard has for a number of years been engaged in the study of electro-therapeutics, and is one of the authors of that most excellent work, "Medical and Surgical Electricity." We have great confidence in his opinions, and with him believe that great results may be expected from this department of science. The following extract will show the object of the author in this publication:

"I desire to call attention to a new department of science—the electro-therapeutics of dermatology. Although the department is yet in its infancy, it is proper that it should now be introduced to the profession, and by preference to those who are especially interested in the study of diseases of the skin—for the two-fold reason, that, young as it is, it gives promise of rapid and useful growth, and because the coöperation of more laborers is needed to make its growth healthful and to bring it to a full and strong maturity."

We also find upon our table, and have been interested in glancing over, two other pamphlets—reprints from the *Louisville and Richmond Medical Journal.* One is from the pen of W. O. Baldwin, M.D., of Montgomery, Alabama, and entitled, "Irrigations of Ice-Water as a Means of Arresting Hemorrhage in Cases of Placenta Prævia." This paper will justly add to the well-known reputation of the author. The other is from J. S. Weatherly, M.D., of the same city, upon medical education, and is an address delivered before the State Medical Association, at Mobile, Alabama.

The Doctor's address is an interesting one, and he deserves credit for his efforts to elevate the standard of medical education. We agree with him fully, that there can be no possible reason for creating new facilities for entrance into the medical profession. Surely, physicians are not in demand, unless it be those who are more thoroughly acquainted with what they profess to practice than the large mass of those who are annually turned out from the medical colleges of the land. Truly, nothing can be more absurd than what is called medical education in the United States, and we see no hope of radical improvement, without securing a higher degree of preliminary education, and separating the teaching and licensing power.

THE LONDON WATER-SUPPLY.—The people of the British metropolis appear to be considerably exercised concerning the water which is supplied to them for drinking and for other domestic purposes. The anxiety is not concerning the quantity of the supply, as was recently the case with our own city; but the filthy quality of the Thames river-water is becoming unendurable, containing, as it does, an abundance of impurities, the washings of dirty London air and of dirtier surface drainage.—*Boston Medical and Surgical Journal.*

Page(s) missing

